# Povidone-iodine preprocedural rinse—An evidence-based, second-line defense against severe acute respiratory coronavirus virus 2 (SARS-CoV-2) in dental healthcare

**DOI:** 10.1017/ice.2021.90

**Published:** 2021-03-12

**Authors:** Raj Kumar Maurya, Harpreet Singh, Pranav Kapoor, Poonam Sharma, Dhirendra Srivastava

**Affiliations:** 1 Central Government Dental Unit, Nagaland, India; 2 Department of Orthodontics & Dentofacial Orthopedics, ESIC Dental College & Hospital, New Delhi

*To the Editor*—Ever since it began, coronavirus disease 2019 (COVID-19) pandemic has brought disruptions to almost all aspects of society, with far-reaching adverse impacts on clinical and economic fronts. Even amid effective vaccine rollout, which has rekindled the hope of ending this unprecedented global disaster, the COVID-19 pandemic continues unabated, with reports of a highly transmissible, mutant, severe acute respiratory coronavirus virus 2 (SARS-CoV-2) VOC 202012/01 strain and another SARS-CoV-2 20H/501Y.V2 variant. These strains could drive even larger waves of disease.^1^ Due to high viral loads and the consequent transmission potential of asymptomatic and minimally symptomatic patients,^2^ reducing the viral load in the oropharynx with adequate oral prophylactic measures is imperative to contain and prevent the spread of SARS-CoV-2 in public and dental healthcare settings. Considering the high-risk nature of the dental healthcare occupation due to the propensity for close-contact transmission through infective saliva, droplet splatter, and blood-mixed aerosols, procedural mitigation strategies involving prophylactic use of preprocedural mouth rinses (PPMRs) has been recommended to attenuate nosocomial transmission of SARS-CoV-2.^3^ Different oral antiseptic agents have been employed as a second layer of defence against microbial load in dental aerosols: povidone-iodine (PVP-I), chlorhexidine gluconate (CHX), hydrogen peroxide, cetylpyridinium chloride (CPC), and essential oils.^4^ A comparative in vitro study evaluating virucidal efficacy of different oral rinses demonstrated that mouth formulations containing 1% PVP-I, combination of dequalinium chloride and benzalkonium chloride, and a combination of ethanol and essential oils can significantly reduce SARS-CoV-2 viral infectivity within short exposure times of 30 seconds.^5^ However, CHX-based mouth rinse exhibited weak virucidal efficacy. A recent systematic review found no scientific evidence supporting the virucidal activity of hydrogen peroxide mouthwash.^6^ With the growing evidence amid the COVID-19 pandemic, evidence-based recommendations in favor of PVP-I as PMR have been increasing.

PVP-I or iodopovidone has been ubiquitously used as an antiseptic in healthcare settings for decades. Comprising iodine and the water-soluble polymer polyvinylpyrrolidone, PVP-I is considered favorable for its slow and gradual iodine release, minimizing toxicity, and for the resultant viral inactivation arising from its oxidative effect and lipid shell-membrane destruction.^7^ Regarding the virucidal activity of PVP-I against SARS-CoV-2, Bidra et al^8^ were among the first researchers to provide direct evidence of rapid in vitro virucidal action of 0.5% PVP-I oral rinse at the lowest contact time of 15 seconds. These researchers also showed that 70% ethanol-based rinse requires a minimum contact time of 30 seconds rather than 15 seconds to completely inactivate SARS-CoV-2. Subsequent in vitro studies conducted by Hassandarvish et al^9^ demonstrated potent and rapid virucidal efficacy of 0.5% and 1% PVP-I rinse at the lowest contact time of 15 seconds, and Anderson et al^10^ also demonstrated the efficacy of 1.0% w/v PVP-I oral rinse and 0.45% w/v PVP-I throat spray with a contact time of 30 seconds.

In real-time hospital settings, Martinez et al^11^ reported significant reduction in SARS-CoV-2 viral load after rinsing with 1% PVP-I for 1 minute, with 3-hourly sustained clinical effects. A recently published sole randomized controlled trial involving 36 SARS-CoV-2–positive patients evaluated the in vivo efficacy of 3 commercial mouth rinses compared with water: PVP-I, CHX, and CPC.^12^ Rinsing with 0.075% CPC and 0.5% PVP-I for 30 seconds decreased the salivary SARS-CoV-2 levels within 5 minutes of use, and the subsequent effects were sustained at 6-hour time points.

No cytotoxic effects of 1% PVP-I mouthwash have been reported when used at a concentration of 0.63 mg/mL (ie, 1:16 or lower dilution of the product).^9^ Moreover, in addition to a well-established safety profile, PVP-I also exhibits good tolerability at concentrations as high as 2.5% for up to 5 months, with the absence of tooth or tongue discoloration or taste disturbances.^13^ Unlike alcohol-based mouth rinse, it can also be used in conjunction with electrocautery, which also make it a suitable choice for use in maxillofacial surgery. Few contraindications for the use of PVP-I solutions include type 1 anaphylactic allergy to iodine (rarely encountered), active thyroid disease, pregnancy, and radioactive iodine therapy. Hence, proper medical and drug history should be reviewed before their use.

In view of the fluid situation posed by the ongoing pandemic, Kirk-Bailey et al^14^ recommend the use of 9 mL 0.5% PVP-I as a mouthwash and 0.28–0.3 mL 0.5% PVP-I as a nasal spray solution in each nostril, both for the patient before examination and/or treatment, and for the clinical staff prior to patient contact (repeated every 2–3 hours, up to 4 times a day, if multiple patients are seen). Due to the chemical instability of PVP-I with respect to disproportionation into constituent equilibrium species, freshly prepared dilutions should be used, and they should be preserved in the refrigerator for subsequent patients through the day.^8^

While reconciling the fact that postpandemic resurgences may occur as late as 2024,^15^ safeguarding the oral healthcare workforce and patients becomes even more pertinent considering the perceived vulnerability to the SARS-CoV-2 infection in dental settings. With a growing body of direct evidence consistently supporting the effectiveness of PVP-I against SARS-CoV-2, it would be reasonable to prioritize the use of PVP-I as an effective and adjunctive procedural risk mitigation measure in dental healthcare in the contemporary pandemic crisis and through unforeseen similar pandemic threats.
